# Enhancement of lactate fraction in poly(lactate-*co*-3-hydroxybutyrate) biosynthesized by metabolically engineered *E. coli*

**DOI:** 10.1186/s40643-024-00803-2

**Published:** 2024-09-19

**Authors:** Binghao Zhang, Pengye Guo, Xinye Sun, Yanzhe Shang, Yuanchan Luo, Hui Wu

**Affiliations:** 1 State Key Laboratory of Bioreactor Engineering, Shanghai Frontiers Science Center of Optogenetic Techniques for Cell Metabolism, School of Biotechnology, East China University of Science and Technology, 130 Meilong Road, Shanghai, 200237 China; 2 MOE Key Laboratory of Bio-Intelligent Manufacturing, School of Bioengineering, Dalian University of Technology, Dalian, China; 3 Shanghai Collaborative Innovation Center for Biomanufacturing Technology, 130 Meilong Road, Shanghai, 200237 China; 4 Key Laboratory of Bio-based Material Engineering of China, National Light Industry Council, 130 Meilong Road, Shanghai, 200237 China

**Keywords:** Poly(lactate-*co*-3-hydroxybutyrate), Lactyl-CoA, CoA transferases, Lactate-based copolymers

## Abstract

Poly(lactate-*co*-3-hydroxybutyrate) [P(LA-*co*-3HB)] is a high-molecular-weight biomaterial with excellent biocompatibility and biodegradability. In this study, the properties of P(LA-*co*-3HB) were examined and found to be affected by its lactate fraction. The efficiency of lactyl-CoA biosynthesis from intracellular lactate significantly affected the microbial synthesis of P(LA-*co*-3HB). Two CoA transferases from *Anaerotignum lactatifermentans* and *Bacillota bacterium* were selected for use in copolymer biosynthesis from 11 candidates. We found that *cot*_*Al*_ enhanced the lactate fraction by 31.56% compared to that of the frequently used modified form of propionyl-CoA transferase from *Anaerotignum propionicum*. In addition, utilizing xylose as a favorable carbon source and blocking the lactate degradation pathway further enhanced the lactate fraction to 30.42 mol% and 52.84 mol%, respectively. Furthermore, when a 5 L bioreactor was used for fermentation utilizing xylose as a carbon source, the engineered strain produced 60.60 wt% P(46.40 mol% LA-*co*-3HB), which was similar to the results of our flask experiments. Our results indicate that the application of new CoA transferases has great potential for the biosynthesis of other lactate-based copolymers.

**Graphical Abstract:**

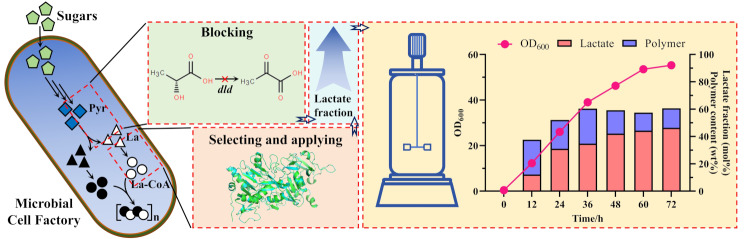

**Supplementary Information:**

The online version contains supplementary material available at 10.1186/s40643-024-00803-2.

## Introduction

Bioplastics, which are biodegradable and biocompatible, offer a promising solution to mitigate increasingly severe resource and environmental challenges posed by fossil-fuel derived plastics (Ali et al. 2024; Choi et al. 2020; Guo et al. 2021; Liao et al. 2024; Lv et al. 2022; Zhong et al. 2023). Among bioplastics, polyhydroxyalkanoates (PHAs) are at the forefront of bioplastics research and development efforts (Ganesh Saratale et al. 2021; Park et al. 2024; Yukesh Kannah et al. 2022). PHAs can be synthesized by microorganisms to achieve extremely high molecular weights at a low cost (Medeiros Garcia Alcântara et al. 2020).

Among the PHA biosynthetic pathways, poly(3-hydroxybutyrate) (PHB) is the most extensively studied (Choi et al. 2020; Ganesh Saratale et al. 2021; Yukesh Kannah et al. 2022). Introducing a lactate monomer into the PHB chain enables the formation of poly(lactate-*co*-3-hydroxybutyrate) [P(LA-*co*-3HB)] and effectively enhances the properties of the polymer. These enhanced properties are dependent on the lactate fraction (Yamada et al. 2011). Microbial synthesis of lactate-based PHAs involves two key processes: the efficient conversion of lactate into lactyl-CoA, and the selection of a PHA synthase that can accept lactyl-CoA as the substrate (Dundas and Dinneny 2022; Taguchi et al. 2008). Significant efforts have been made to achieve the first goal.

P(LA-*co*-3HB) was biosynthesized for the first time using propionyl-CoA transferase (Pct) from *Megasphaera elsdenii* (Taguchi et al. 2008). Subsequently, Pct from *Anaerotignum propionicum*, due to its toxicity and inefficient conversion of lactate into lactyl-CoA, was modified to generate Pct532_*Ap*_ and Pct540_*Ap*_ (Yang et al. 2010). Pct_*Me*_ and Pct540_*Ap*_ have been widely used to produce various lactate-based copolymers (Choi et al. 2016; Li et al. 2016, 2017). Pcts from *Clostridium perfringens* (Jin et al. 2016) and several butyryl-CoA transferases (Bcts) (David et al. 2017) have also been used. The production of other products derived from the conversion of lactate into lactyl-CoA, including propionate (Balasubramanian et al. 2020; Baur et al. 2022; Kandasamy et al. 2013), 1,2-propanediol (Niu and Guo 2015; Niu et al. 2019), lactate esters (Lee and Trinh 2019; Ren et al. 2020), and polylactate (PLA) (Lajus et al. 2020; Shi et al. 2022; Tan et al. 2022; Ylinen et al. 2021), uses Pcts from *A. neopropionicum* (Baur et al. 2022), *Cupriavidus necator* (Lajus et al. 2020; Ren et al. 2020), and *Moorella thermoacetica* (Ren et al. 2020). In addition, acyl-CoA: acetate/3-ketoacid CoA transferase from *Megasphaera* sp. DISK 18 (Zhang et al. 2019), 3-ketoacid CoA transferase from *A. lactatifermentans* (Zhang et al. 2019), and acetate CoA transferase from *Bacillota bacterium* (Zhang et al. 2019) and *Escherichia coli* (*ydiF*) (Dong et al. 2022) have the ability to convert CoA to lactate.

The composition of P(LA-*co*-3HB) can be affected by the intracellular lactyl-CoA concentration, as it influences the mobility of the polymerized product (Matsumoto et al. 2018). The enzyme activity of the converting lactate into lactyl-CoA affects the composition of the copolymer. Therefore, we tested various CoA transferases in the copolymer biosynthesis system in order to select enzymes that are more conducive to the copolymerization of the high-lactate fraction. In addition, multiple strategies were used to further amplify the advantages of the selected enzymes. Finally, in addition to CoA transferases, several CoA synthetases were tested to determine whether CoA can be directly linked to lactate.

## Methods

### Strains and plasmids

The strains and plasmids used in this study are listed in Table S1. Strain WXJ01 and plasmids pTrc99aABC and pBad33-P*trc*-*pct540*_*Ap*_ were obtained from previous studies (Lu et al. 2019; Wei et al. 2021; Yang et al. 2010). CoA transferases and synthetases from different sources were codon optimized, synthesized, and inserted into pBad33-P*trc* using gene synthesis and plasmid DNA preparation services (GenScript, Nanjing, China). The original promoter of pBad33 was replaced with the IPTG-induced *trc* promoter (pTrc99a source) to form pBad33-P*trc.* The plasmids were transformed into strains by electroporation.

### Medium and culture conditions

Luria-Bertani medium with 10 g NaCl, 10 g tryptone, and 5 g yeast extract per liter was used for the seed culture. Fermentation medium with 15.12 g Na_2_HPO_4_·12H_2_O, 3 g KH_2_PO_4_, 0.5 g NaCl, 1 g NH_4_Cl, 0.493 g MgSO_4_·7H_2_O, 0.0111 g CaCl_2_, and 0.002 g vitamin B1 per liter was used for the shake flask culture. Ampicillin (100 mg/L) and chloramphenicol (34 mg/L) were added to ensure plasmid stability, and the seed was cultured at 37℃ and 220 rpm. A 2% (v/v) seed was added to shaker flask and cultured overnight. The shake flask was cultured at 30℃ and 220 rpm and allowed to ferment for 60 h. For the substrate, 10 g/L glucose or xylose was added. IPTG (0.1 mM) was added at the beginning of the fermentation process to induce enzyme expression. All shake flask experiments were performed in triplicates.

### Fed-batch fermentation

The 5 L bioreactor experiment was performed according to previously described instrumentation and inoculation methods (Wu et al. 2023). The bioreactor medium contained 50 g/L xylose and 5 g/L yeast extract. The culture temperature was maintained at 30 °C and the pH was maintained at 7.0 by using NH_4_OH (25%, v/v). Feeding was set at 36 h, and the xylose concentration was maintained at 50 g/L.

### Analytical methods

Optical density was measured at 600 nm. The concentrations of glucose, xylose, and lactate were measured using high-performance liquid chromatography (HPLC) (LC-20 A, Shimadzu, Japan) with a refractive index detector (RID-20 A, Shimadzu, Japan) and cation exchange column (HPX-87 H, Bio-Rad, United States). The detector temperature was 45℃ and the column temperature was 65℃. For the mobile phase, 5 mM H_2_SO_4_ was used at a flow rate of 0.6 mL/min. The intracellular polymer content and fraction of lactate in the copolymer after 60 h were determined using a previously described method (Wu et al. 2021, 2023).

## Results and discussion

### Selecting and applying CoA transferases and synthetases for P(LA-*co*-3HB) production

The Pct from *C. necator* was not considered a good choice because of its poor specificity (Lindenkamp et al. 2013; Volodina et al. 2014). The catalytic efficiency (*k*_cat_/*K*_m_) of the CoA transferase from *Megasphaera* sp. DISK 18 was lower than that of *cot*_*Al*_ and *cot*_*Bb*_ (Zhang et al. 2019). Thus, *cot*_*Al*_, *cot*_*Bb*_, and *ydiF* were selected, which are all members of the OXCT1 family (Hackmann 2022) of CoA transferases. We also selected other enzymes, including *cot*_*Dm*_, *cot*_*Se*_, and *cot*_*Sc*_. With the exception of *cot*_*Sc*_, which has DXGXXG and GXGG(A/F) motifs, all of the other selected enzymes contain the highly conserved EXGXXG and GXGG(A/F) sequence motifs (Fig. S1) (Rangarajan et al. 2005). The identical sequence motifs show that these six enzymes belong to the same enzyme family, indicating the possibility of the latter three (*cot*_*Dm*_, *cot*_*Se*_, and *cot*_*Sc*_) catalyzing lactate.

In addition, several CoA synthetases were selected based on their ability to catalyze the production of short-chain fatty acids (acetate, propionate, and butyrate) (Yoshimura et al. 2017). It has been speculated that the active pocket of these enzymes accepts lactate (2-hydroxypropionate) to generate lactyl-CoA. Compared to acyl-CoA synthetase short-chain (ACSS) family members ACSS1 and ACSS2, the preferred substrate of ACSS3 is propionate (Yoshimura et al. 2017). Thus, ACSS3_*Mm*_ (Wang et al. 2024) and ACSS3_*Hs*_, which is 89.50% identical to ACSS3_*Mm*_ (Fig. S2), were selected. Acetoacetate is the simplest 3-ketoacid, and *cos*_*Pa*_, which uses acetoacetate as a substrate, may catalyze lactate. Therefore, the effect of *cos*_*Pa*_ was also tested. Previous studies have shown that propionyl-CoA synthetase (*prpE*) from *E. coli* cannot catalyze the conversion of lactate to lactyl-CoA (data not shown). However, the effect of medium-chain fatty acid CoA synthetase (*fadK*) from *E. coli*, which prefers C6-C8 chain fatty acid substrates (Morgan-Kiss and Cronan 2004), was tested as well as *cos*_*Sf*_, which is 97.35% identical to *fadK* (Fig. S3).

**Fig. 1:**
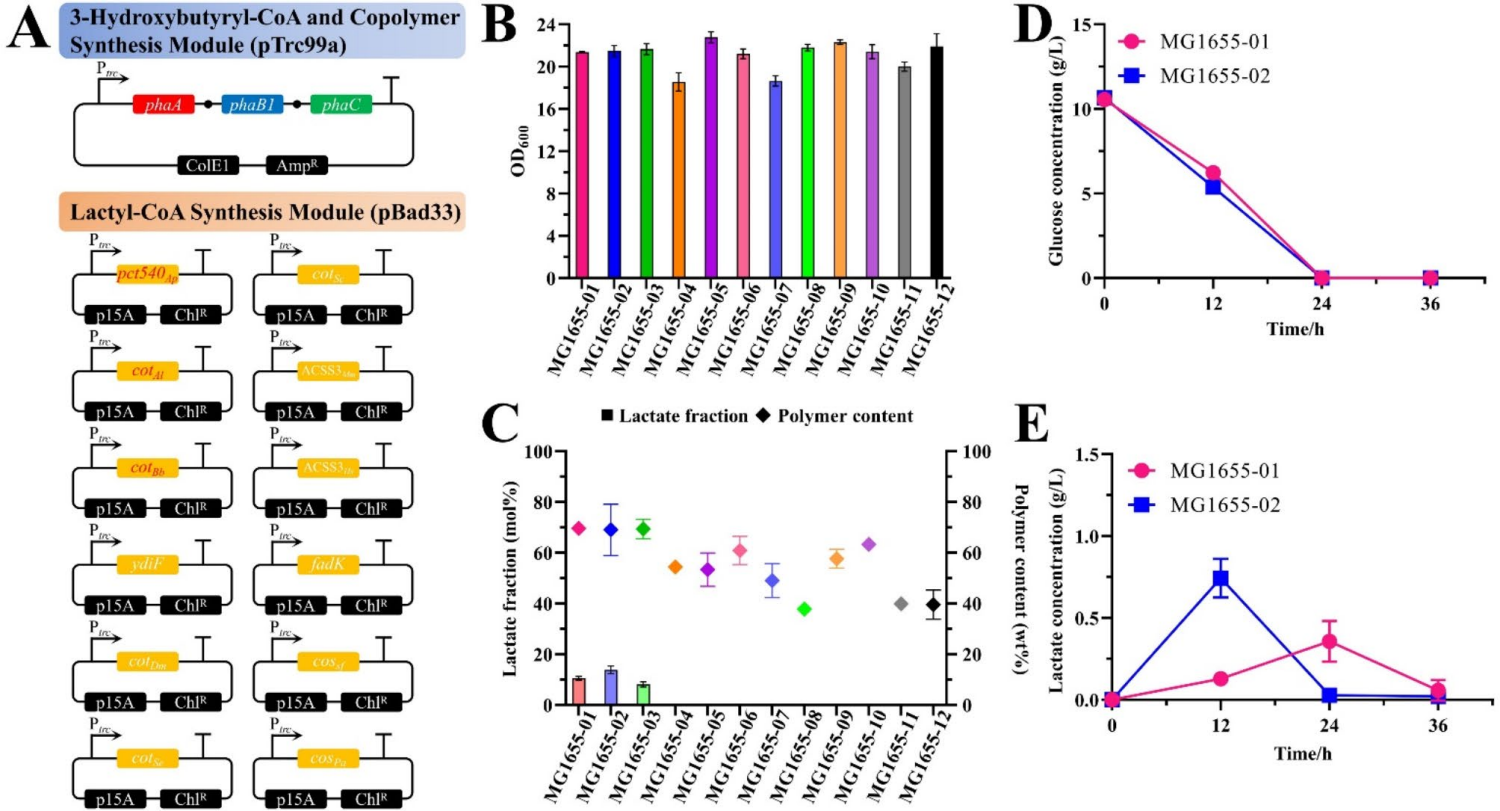
P(LA-*co*-3HB) biosynthesis of MG1655 using CoA transferases and synthetases from different sources with 10 g/L glucose. **A**, the schematic diagrams of 3-hydroxybutyrate-CoA and copolymer synthesis module and lactyl-CoA synthesis module [the red-labeled enzymes in pBad33-P*trc* can efficiently provide lactyl-CoA required for P(LA-*co*-3HB) biosynthesis]; **B**, the optical density at 60 h; **C**, the P(LA-*co*-3HB) content and the lactate fraction; **D**, the glucose consumption; **E**, the lactate production

The pre-constructed plasmids (Fig. 1A) were transformed into *E. coli* and *pct540*_*Ap*_ was used as a control to verify the effect of the selected 11 enzymes. Results of MG1655-01–12 using glucose are shown in Fig. 1B and C. Only *cot*_*Al*_ and *cot*_*Bb*_ added the lactate fraction to the polymer, which produced 69.00 wt% P(13.88 mol% LA-*co*-3HB) and 69.35 wt% P(8.12 mol% LA-*co*-3HB) respectively. Of these, only the effect of *cot*_*Al*_ was better than that of *pct540*_*Ap*_ [69.65 wt% P(10.55 mol% LA-*co*-3HB)].

Although *cot*_*Al*_ and *cot*_*Bb*_ had better *k*_cat_/*K*_m_ values than that of *pct540*_*Ap*_ (Zhang et al. 2019), *cot*_*Bb*_ did not demonstrate an advantage in the copolymer biosynthesis system. These results may be due to the differences in the in vitro and in vivo activities of these enzymes. The application of *cot*_*Al*_ increased the lactate concentration (Fig. 1E), indicating that the large flux of *cot*_*Al*_ drives the carbon flow toward lactate, thereby producing a copolymer containing a higher lactate fraction. The glucose consumption rate of MG1655-02 increased slightly (Fig. 1D), possibly because the increased lactate overflow enahnced carbon source utilization (Wei et al. 2021). The protein structures of *pct540*_*Ap*_ and *cot*_*Al*_ are highly similar (Fig. S4), confirming the ability of *cot*_*Al*_ to catalyze the conversion of lactate into lactyl-CoA. Therefore, a copolymer biosynthesis system containing *cot*_*Al*_ was the starting point for subsequent research.

### Xylose enhances the lactate fraction in the P(LA-*co*-3HB) copolymer

**Fig. 2:**
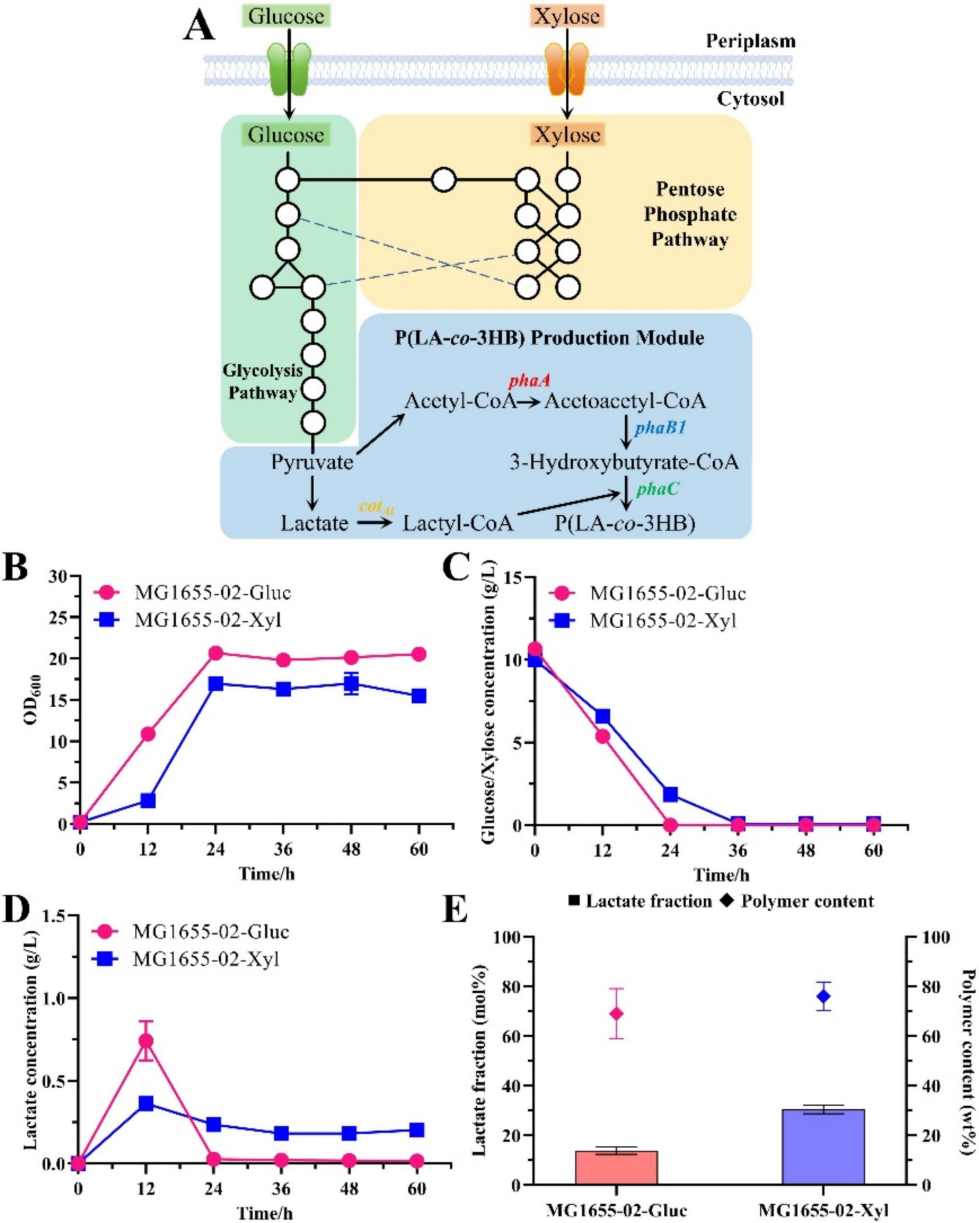
P(LA-*co*-3HB) biosynthesis of MG1655-02 with 10 g/L glucose or xylose. **A**, the metabolic pathway of P(LA-*co*-3HB) biosynthesis from glucose or xylose (solid lines represent reactions, blank circles represent compounds, and dotted lines connect same compounds at both ends); **B**, the optical density; **C**, the substrate consumption; **D**, the lactate production; **E**, the P(LA-*co*-3HB) content and the lactate fraction

Compared to glucose, xylose utilization enhanced the lactate fraction in the copolymer (Nduko et al. 2013; Wu et al. 2023). When xylose was used, MG1655-02 produced 76.03 wt% P(30.42 mol% LA-*co*-3HB) with a significantly higher lactate fraction compared to that of glucose (Fig. 2A and E).

The final optical density of MG1655-02 using xylose was lower than that of MG1655-02 using glucose (Fig. 2B), which is consistent with the slower substrate consumption rate of xylose (Fig. 2C). This phenomenon has been observed in a previous study (Wu et al. 2023) and may be caused by lower energy production and higher CO_2_ release from xylose metabolism (Gonzalez et al. 2017). Although the total production of NADH and NADPH was similar for both sugars, the xylose utilization pathway via the pentose phosphate pathway and the greater tricarboxylic acid (TCA) cycle flux of xylose caused xylose to produce NADH and NADPH at a different production source ratio than glucose (Gonzalez et al. 2017), which may explain why the copolymer produced from xylose contained a higher lactate fraction.

### Blocking the D-lactate degradation pathway improves the lactate fraction in the P(LA-*co*-3HB) copolymer

**Fig. 3:**
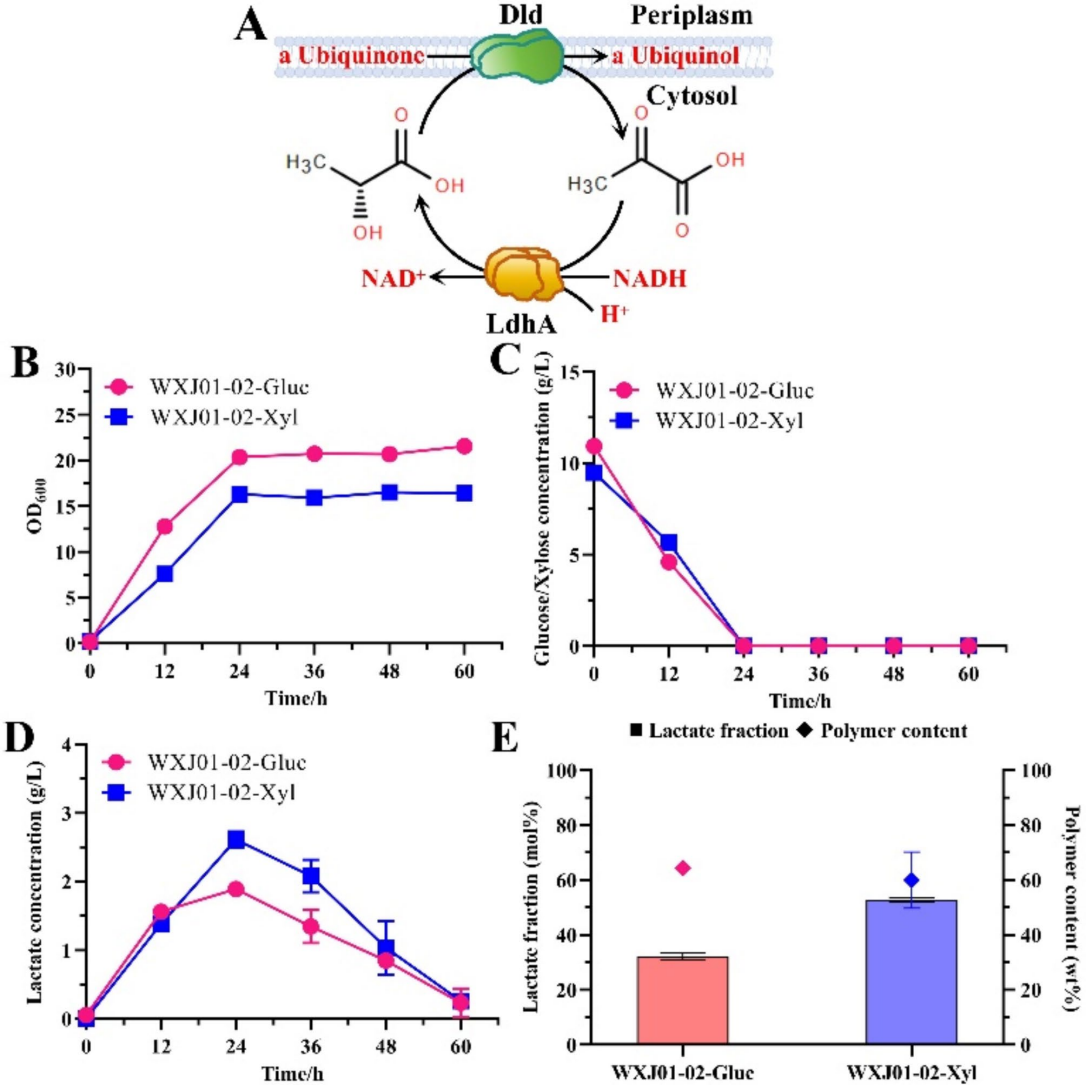
P(LA-*co*-3HB) biosynthesis of WXJ01-02 with 10 g/L glucose or xylose. **A**, the schematic diagram of the intracellular conversion of D-lactate and pyruvate; **B**, the optical density; **C**, the substrate consumption; **D**, the lactate production; **E**, the P(LA-*co*-3HB) content and the lactate fraction

In *E. coli*, quinone-dependent D-lactate dehydrogenase (*dld*) converts D-lactate into pyruvate (Fig. 3A) (Dym et al. 2000). It is believed that knocking out *dld* causes the accumulation of lactate and the formation of more lactyl-CoA, thereby enhancing the lactate fraction in the copolymer (Choi et al. 2016; Lu et al. 2019; Nduko et al. 2014; Wei et al. 2021; Wu et al. 2021). WXJ01-02 produced 64.38 wt% P(32.08 mol% LA-*co*-3HB) from glucose and 59.93 wt% P(52.84 mol% LA-*co*-3HB) from xylose (Fig. 3E).

Xylose was found to be better than glucose as carbon sources for P(LA-*co*-3HB) biosynthesis. The final optical density of glucose was still higher than that of xylose (Fig. 3B), which is similar to the results in the previous section (Fig. 2B). Both sugars were utilized at slightly faster rates (Figs. 2C and 3C). The increase in carbon source utilization may be due to the fact that lactate cannot enter the TCA cycle via pyruvate after lactate reflux is blocked (Wei et al. 2021). The lactate concentration peaked at 24 h (Fig. 3D) and was significantly higher compared to that without *dld* deletion (Fig. 2D), which explains why the lactate fraction in the copolymer was significantly enhanced with *dld* deletion.

We explored the effects of *dld* deletion on cell metabolism and physiology without introducing the copolymer synthesis module. The *dld* deletion only significantly increased lactate production without affecting cell growth, substrate consumption, and acetate production (Fig. S5A-H). This further demonstrates that *dld* deletion is an effective strategy to increase the lactate fraction in the copolymer. Environmental acidification caused by a high acetate concentration and the toxicity of acetate (Chun et al. 2014) inhibited cell metabolism (Fig. S5B and D). The introduction of the copolymer synthesis module can significantly pull the carbon flow toward lactate (Fig. 3D and Fig. S5C and G).

### P(LA-*co*-3HB) biosynthesis using the bioreactor

**Fig. 4:**
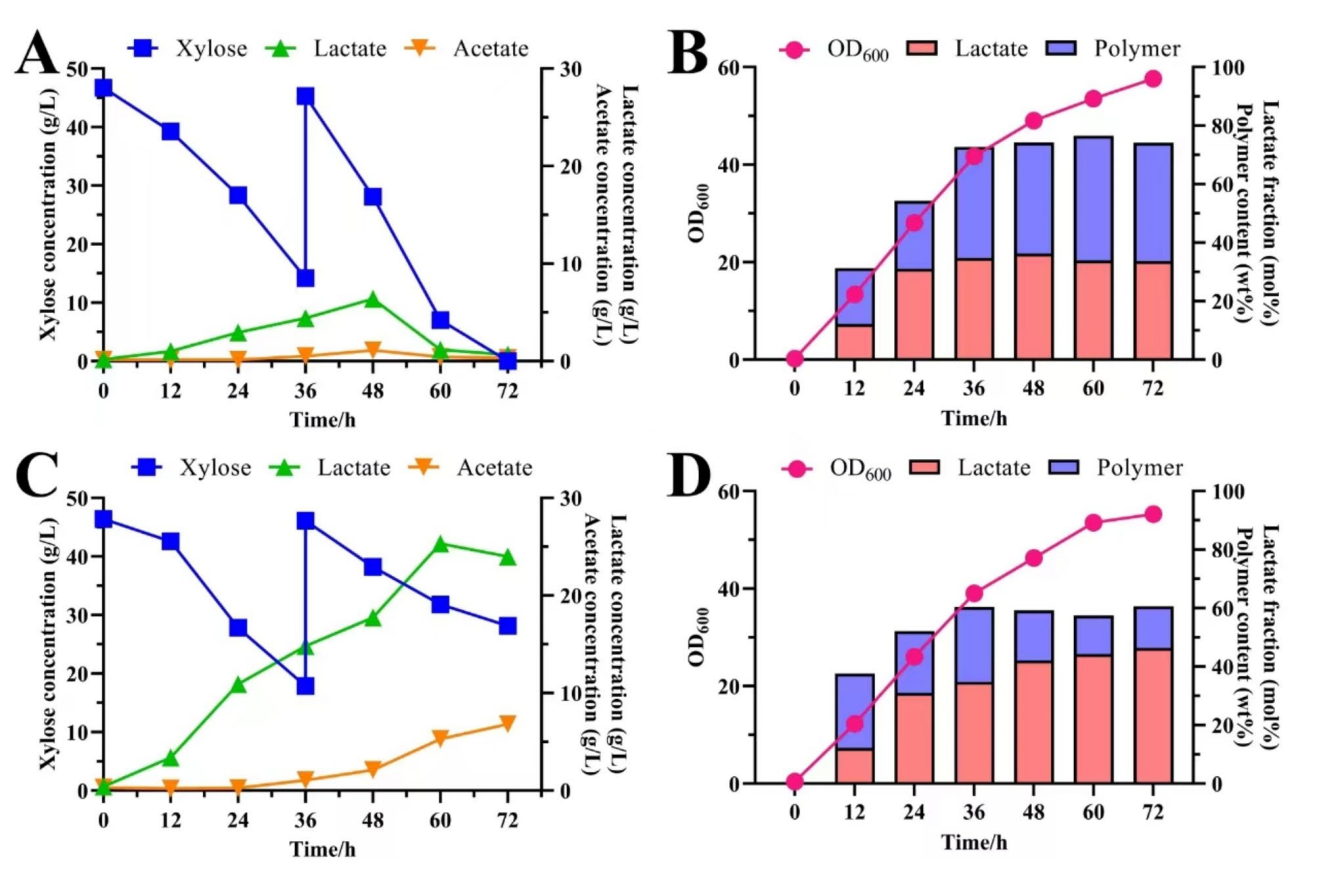
P(LA-*co*-3HB) biosynthesis of MG1655-02 and WXJ01-02 with 10 g/L xylose in a 5 L bioreactor. **A**, the xylose consumption and the lactate and the acetate production of MG1655-02; **B**, the optical density, the P(LA-*co*-3HB) content, and the lactate fraction of MG1655-02; **C**, the xylose consumption and the lactate and the acetate production of WXJ01-02; **D**, the optical density, the P(LA-*co*-3HB) content, and the lactate fraction of WXJ01-02

The effects of MG1655-02 and WXJ01-02 were scaled up in a 5 L bioreactor. The two strains eventually produced 74.14 wt% P(33.73 mol% LA-*co*-3HB) and 60.60 wt% P(46.40 mol% LA-*co*-3HB) respectively, and their polymer contents reached a stable level at 36 h (Fig. 4B and D), which indicates that xylose did not flow into the copolymer after feeding.

MG1655-02 consumed all xylose at 72 h, whereas WXJ01-02 had 28.15 g/L of xylose remaining (Fig. 4A and C). However, the optical densities ​​of both strains were similar (Fig. 4B and D), indicating that xylose did not flow into the biomass after feeding. No obvious byproducts except lactate and acetate were detected using HPLC. However, MG1655-02 produced more bubbles than WXJ01-02 after feeding during fermentation, indicating that MG1655-02 converted xylose into gas (possibly CO_2_ or H_2_). WXJ01-02 converted xylose into lactate and acetate (Fig. 4C). In contrast to the results obtained in the shake flask (Figs. 2C and 3C), the xylose consumption rate of WXJ01-02 was much lower than that of MG1655-02 (Fig. 4A and C). This may be because a high lactate concentration is toxic to cells (Chun et al. 2014), thus affecting the metabolism of WXJ01-02. At 48 h, MG1655-02 showed obvious consumption of lactate (no flow into the copolymer) and acetate, and this process began with the massive consumption of xylose (Fig. 4A), indicating the existence of a non-strict hierarchical carbon utilization (Okano et al. 2021). The polymer content of MG1655-02 was higher than that of WXJ01-02, whereas the lactate fraction of MG1655-02 was lower than that of WXJ01-02 (Fig. 4B and D). The incorporation of lactyl-CoA in the polymer chain leads to premature termination of the polymer chain (Matsumoto et al. 2018); thus, the higher the lactate fraction, the lower the polymer content. The lactate fraction of WXJ01-02 increased over time. However, the polymer content did not change significantly (Fig. 4D), indicating that the produced polymer was not uniform. When the lactate concentration was low, a polymer with a low lactate fraction was produced. When the lactate concentration was high, a polymer with a high lactate fraction was produced. Without affecting the polymer content, the bioreactor increased the lactate fraction of MG1655-02 but decreased the lactate fraction of WXJ01-02. The decreased lactate fraction may be due to the toxicity of lactate and influenced by the lactyl-CoA concentration. Therefore, the lactate concentration in the bioreactor should be strictly controlled.

## Conclusions

Among the selected 11 enzymes, *cot*_*Al*_ and *cot*_*Bb*_ supported P(LA-*co*-3HB) production. Compared with *pct540*_*Ap*_, *cot*_*Al*_ performed better in the copolymer biosynthesis system. Xylose was a more favorable carbon source than glucose. Knockout of *dld* further enhanced the lactate fraction. Ultimately, 59.93 wt% P(52.84 mol% LA-*co*-3HB) was produced in a shake flask. Furthermore, when a 5 L bioreactor was used for fermentation utilizing xylose as a carbon source, the engineered strain produced 60.60 wt% P(46.40 mol% LA-*co*-3HB). The results indicate that the application of new CoA transferases has great potential for the biosynthesis of other lactate-based copolymers.

## Electronic supplementary material

Below is the link to the electronic supplementary material.

Supplementary Material 1

## Abbreviations

PHA: Polyhydroxyalkanoate
PHB: Poly(3-hydroxybutyrate)
P(LA-co-3HB): Poly(lactate-co-3-hydroxybutyrate)
Pct: Propionyl-CoA transferase
Bct: Butyryl-CoA transferase
PLA: Polylactate
ydiF: Acetate CoA transferase from E. coli
HPLC: High performance liquid chromatography
k_cat_/K_m_: The catalytic efficiency
cot_Al_: CoA transferase from A. lactatifermentans
cot_Bb_: CoA transferase from B. bacterium
cot_Dm_: CoA transferase from D. melanogaster
cot_Se_: CoA transferase from Sa. enterica
cot_Sc_: CoA transferase from St. carnosus
ACSS: Acyl-CoA synthetase short-chain
ACSS3_Mm_: CoA synthetase from M. musculus
ACSS3_Hs_: CoA synthetase from H. sapiens
cos_Pa_: CoA synthetase from P. aeruginosa
prpE: Propionyl-CoA synthetase from E. coli
fadK: Medium-chain fatty acid CoA synthetase from E. coli
cos_Sf_: CoA synthetase from Sh. flexneri
TCA: Tricarboxylic acid
dld: Quinone-dependent D-lactate dehydrogenase from E. coli
Amp^R^: Ampicillin resistance
Chl^R^: Chloramphenicol resistance
phaA: Acetyl-CoA acetyltransferase from C. necator
phaB1: Acetoacetyl-CoA reductase from C. necator
phaC: Class II poly(R)-hydroxyalkanoic acid synthase from P. fluorescens
ldhA: D-lactate dehydrogenase from E. coli
