# Correction: Enhancement of lactate fraction in poly(lactate-co-3-hydroxybutyrate) biosynthesized by metabolically engineered *E. coli*

**DOI:** 10.1186/s40643-024-00810-3

**Published:** 2024-10-12

**Authors:** Binghao Zhang, Pengye Guo, Xinye Sun, Yanzhe Shang, Yuanchan Luo, Hui Wu

**Affiliations:** 1 State Key Laboratory of Bioreactor Engineering, Shanghai Frontiers Science Center of Optogenetic Techniques for Cell Metabolism, School of Biotechnology, East China University of Science and Technology, 130 Meilong Road, Shanghai, 200237 China; 2 MOE Key Laboratory of Bio-Intelligent Manufacturing, School of Bioengineering, Dalian University of Technology, Dalian, China; 3 Shanghai Collaborative Innovation Center for Biomanufacturing Technology, 130 Meilong Road, Shanghai, 200237 China; 4 Key Laboratory of Bio-based Material Engineering of China, National Light Industry Council, 130 Meilong Road, Shanghai, 200237 China

**Correction: Zhang et al. Bioresources and Bioprocessing (2024) 11:88**

10.1186/s40643-024-00803-2.

The authors identified that the Table 1 was mistakenly published, as it is response to the reviewers’ comments.

The original article has been corrected.

