# Supplementary material for: Enhancement of lactate fraction in poly(lactate-co-3-hydroxybutyrate) biosynthesized by metabolically engineered E. coli
Source: Bioresour Bioprocess. 2024 Sep 19;11(1):88. doi: 10.1186/s40643-024-00803-2 (PMC11413402; doi:10.1186/s40643-024-00803-2)
Supplement: Supplementary file 1 — Supplementary Material 1 [file 40643_2024_803_MOESM1_ESM.docx]

**Supplementary Information for:**

**Enhancement of lactate fraction in poly(lactate-*co*-3-hydroxybutyrate) biosynthesized by metabolically engineered *E. coli***

Binghao Zhang^1#^, Pengye Guo^1#^, Xinye Sun^1^, Yanzhe Shang^2^, Yuanchan Luo^1^, Hui Wu^1,2,3,4*^

^1^ State Key Laboratory of Bioreactor Engineering, Shanghai Frontiers Science Center of Optogenetic Techniques for Cell Metabolism, School of Biotechnology, East China University of Science and Technology, 130 Meilong Road, Shanghai 200237, China

^2^ MOE Key Laboratory of Bio-Intelligent Manufacturing, School of Bioengineering, Dalian University of Technology, Dalian, China.

^3^ Shanghai Collaborative Innovation Center for Biomanufacturing Technology, 130 Meilong Road, Shanghai 200237, China

^4^ Key Laboratory of Bio-based Material Engineering of China National Light Industry Council, 130 Meilong Road, Shanghai 200237, China

* Corresponding author: Hui Wu

Telephone: +86-21-64252257

Fax: +86-21-64252250

E-mail: [hwu@ecust.edu.cn](mailto:hwu@ecust.edu.cn)

Table S1. The strains and plasmids used in this study.

| **Strains and plasmids** | **Descriptions** | **Sources** |
| --- | --- | --- |
| **Plasmids** |  |  |
| pTrc99a | Cloning vector, Ampicillin resistance (Amp^R^), ColE1 origin, *trc* promoter | Lab collection |
| pBad33 | Cloning vector, Chloramphenicol resistance (Chl^R^), p15A origin, *araBAD* promoter | Lab collection |
| pTrc99aABC | pTrc99a containing the codon-optimized *phaA* and the codon-optimized *phaB1* from *C. necator* H16 and *phaC* (E130D, S325T, Q481K) from *Pseudomonas fluorescens* 2P24 | (Lu et al., 2019) |
| pBad33-P*trc*-*pct540_Ap_* | pBad33 containing *pct* (V193A, T78C, T669C, A1125G, T1158C) from *A. propionicum* DSM 1682 | (Wei et al., 2021) |
| pBad33-P*trc*-*cot_Al_* | pBad33 containing *cot* (3-ketoacid CoA transferase) from *A. lactatifermentans* An75 | This study |
| pBad33-P*trc*-*cot_Bb_* | pBad33 containing *cot* (acetate CoA transferase) from *B. bacterium* CAG:466 | This study |
| pBad33-P*trc*-*ydiF* | pBad33 containing *ydiF* (acetate CoA transferase) from *E. coli* MG1655 | This study |
| pBad33-P*trc*-*cot_Dm_* | pBad33 containing *cot* (succinyl-CoA:3-ketoacid CoA transferase, isoform B) from *Drosophila melanogaster* | This study |
| pBad33-P*trc*-*cot_Se_* | pBad33 containing *cot* (acetate CoA transferase) from *Salmonella enterica* subsp. *enterica* serovar Montevideo str. CDC 2012K-1544 | This study |
| pBad33-P*trc*-*cot_Sc_* | pBad33 containing *cot* (acyl-CoA:acetate/3-ketoacid CoA transferase) from *Staphylococcus carnosus* TMW 2.269 | This study |
| pBad33-P*trc*-ACSS3*_Mm_* | pBad33 containing *cos* (acyl-CoA synthetase short-chain family member 3, mitochondrial isoform 1) from *Mus musculus* | This study |
| pBad33-P*trc*-ACSS3*_Hs_* | pBad33 containing *cos* (acyl-CoA synthetase short-chain family member 3, mitochondrial isoform 2 precursor) from *Homo sapiens* | This study |
| pBad33-P*trc*-*fadK* | pBad33 containing *fadK* (medium-chain fatty acid CoA synthetase) from *E. coli* MG1655 | This study |
| pBad33-P*trc*-*cos_Sf_* | pBad33 containing *cos* (CoA synthetase) from *Shigella flexneri* 2a str. 301 | This study |
| pBad33-P*trc*-*cos_Pa_* | pBad33 containing *cos* (acetoacetate CoA synthetase) from *P. aeruginosa* PAO1 | This study |
| **Strains** |  |  |
| *E. coli* MG1655 | K-12, F, λ, *ilvG*, *rfb-50*, *rph-1*, | Lab collection |
| WXJ01 | MG1655 Δ*dld* | (Wei et al., 2021) |
| MG1655-01 | MG1655 carrying pTrc99aABC and pBad33-P*trc*-*pct540_Ap_* | This study |
| MG1655-02 | MG1655 carrying pTrc99aABC and pBad33-P*trc*-*cot_Al_* | This study |
| MG1655-03 | MG1655 carrying pTrc99aABC and pBad33-P*trc*-*cot_Bb_* | This study |
| MG1655-04 | MG1655 carrying pTrc99aABC and pBad33-P*trc*-*ydiF* | This study |
| MG1655-05 | MG1655 carrying pTrc99aABC and pBad33-P*trc*-*cot_Dm_* | This study |
| MG1655-06 | MG1655 carrying pTrc99aABC and pBad33-P*trc*-*cot_Se_* | This study |
| MG1655-07 | MG1655 carrying pTrc99aABC and pBad33-P*trc*-*cot_Sc_* | This study |
| MG1655-08 | MG1655 carrying pTrc99aABC and pBad33-P*trc*-ACSS3*_Mm_* | This study |
| MG1655-09 | MG1655 carrying pTrc99aABC and pBad33-P*trc*-ACSS3*_Hs_* | This study |
| MG1655-10 | MG1655 carrying pTrc99aABC and pBad33-P*trc*-*fadK* | This study |
| MG1655-11 | MG1655 carrying pTrc99aABC and pBad33-P*trc*-*cos_Sf_* | This study |
| MG1655-12 | MG1655 carrying pTrc99aABC and pBad33-P*trc*-*cos_Pa_* | This study |
| WXJ01-02 | WXJ01 carrying pTrc99aABC and pBad33-P*trc*-*cot_Al_* | This study |


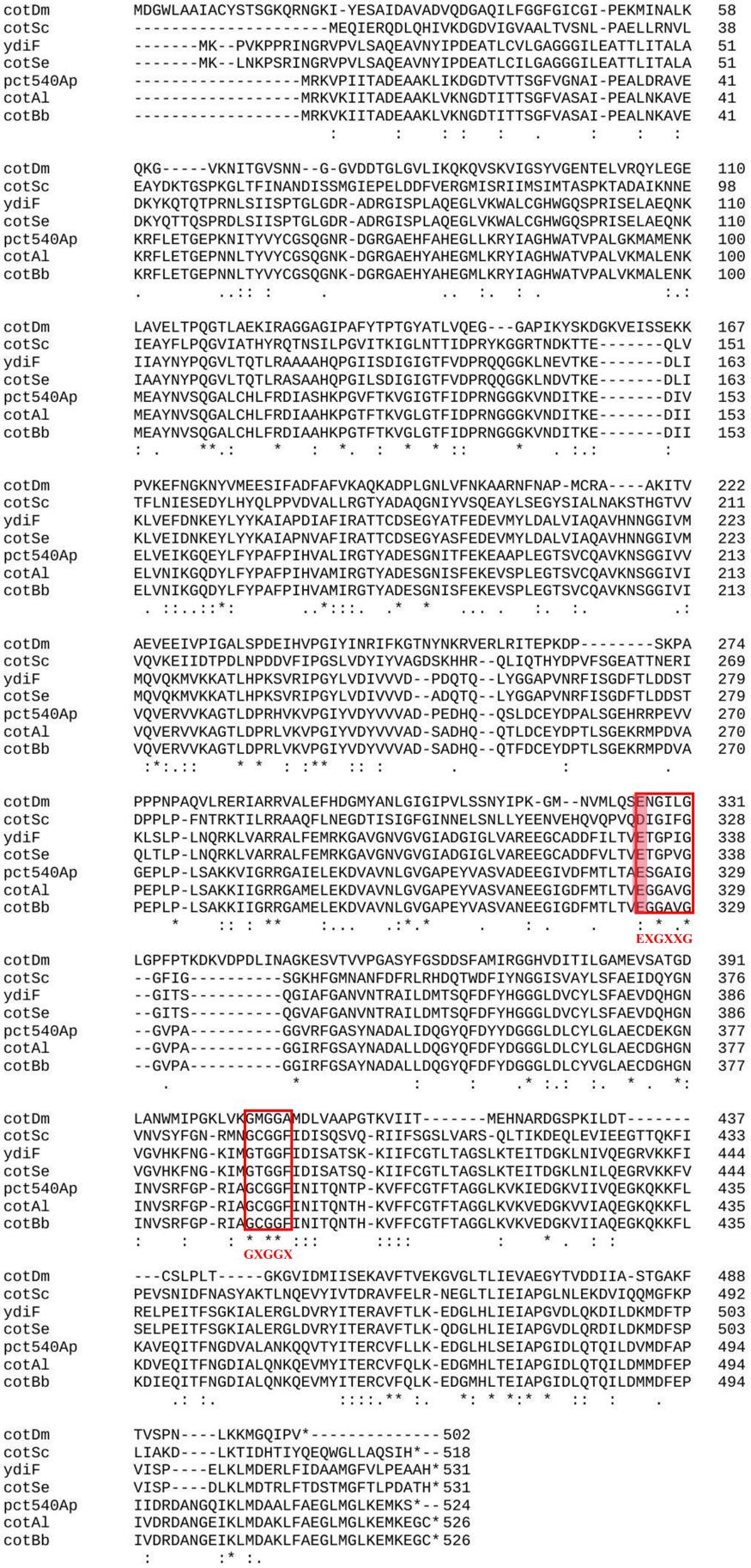


Fig. S1. The amino acid sequence alignment of CoA transferases by Clustal Omega. The highly conserved EXGXXG and GXGG(A/F) sequence motifs are marked by red boxes (*cot_Sc_* is DXGXXG). The catalytic glutamate residue is marked by red shade (*cot_Sc_* is aspartate). It can be seen that *ydiF* and *cot_Se_* are highly homologous, and *pct540_Ap_*, *cot_Al_*, and *cot_Bb_* are highly homologous.


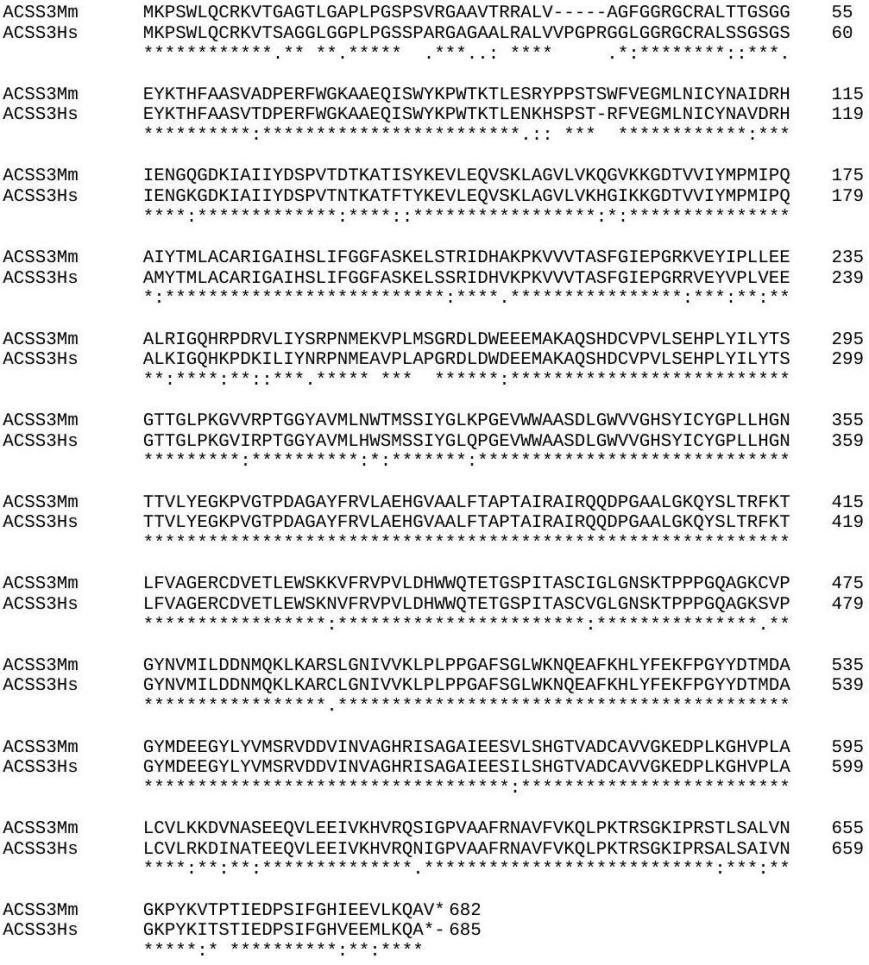


Fig. S2. The amino acid sequence alignment of ACSS3*_Mm_* and ACSS3*_Hs_* by Clustal Omega. It can be seen that the two are highly homologous.


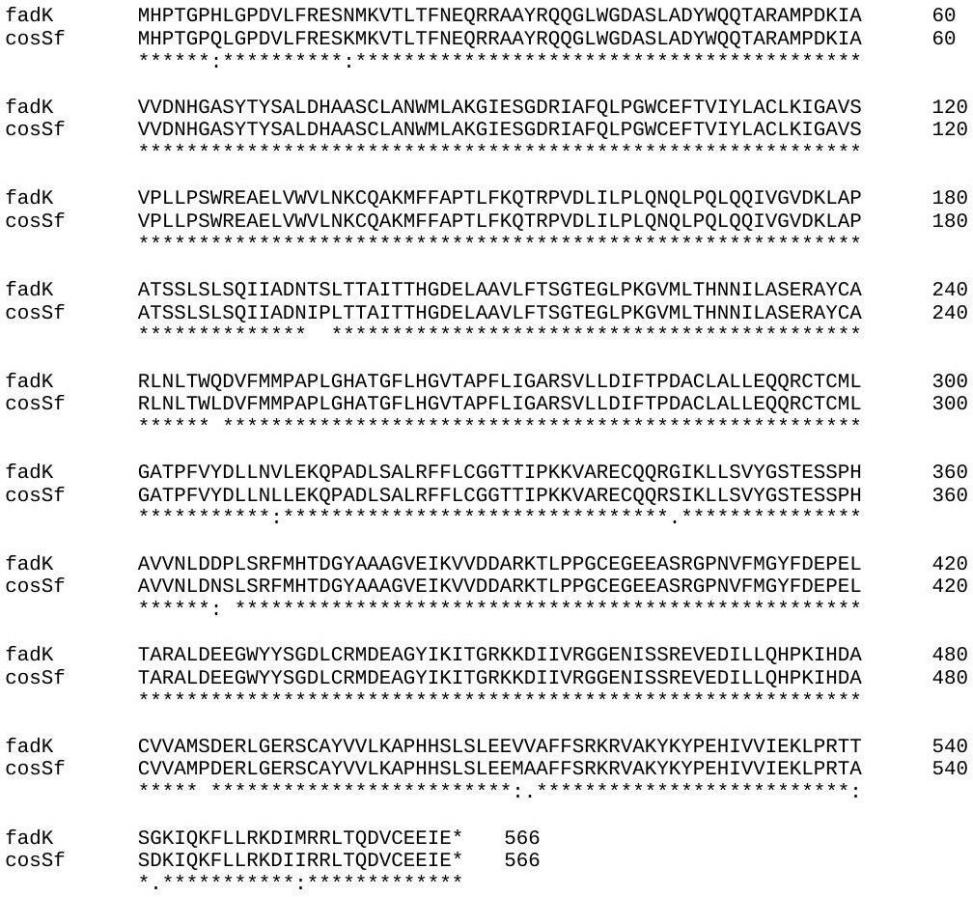


Fig. S3. The amino acid sequence alignment of *fadK* and *cos_Sf_* by Clustal Omega. It can be seen that the two are highly homologous.


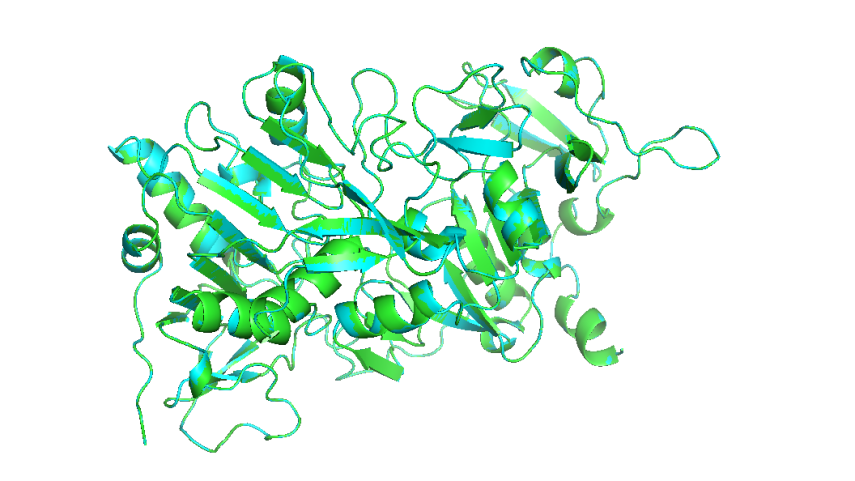


Fig. S4. The comparison of the protein structures of *pct540_Ap_* and *cot_Al_*. Blue is *pct540_Ap_* and green is *cot_Al_*. Swiss-Model is used for the homology modeling and Pymol is used for the comparison of the protein structures.


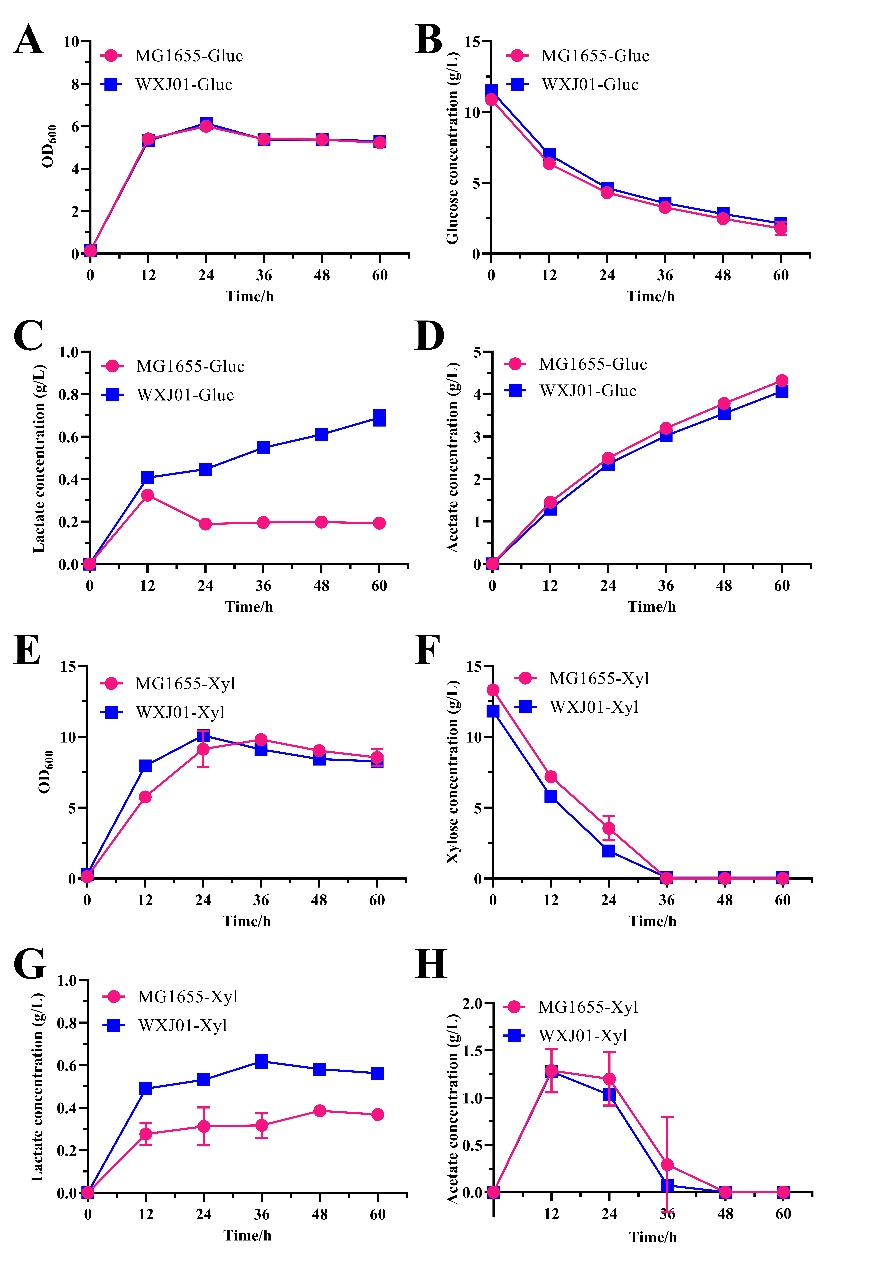


Fig. S5. Cell metabolism and physiology of MG1655 and WXJ01 with 10 g/L glucose or xylose. A, the optical density; B, the glucose consumption; C, the lactate production; D, the acetate production; E, the optical density; F, the xylose consumption; G, the lactate production; H, the acetate production.

**References**

Lu, J., Li, Z., Ye, Q., & Wu, H. (2019). Effect of reducing the activity of respiratory chain on biosynthesis of poly(3-hydroxybutyrate-*co*-lactate) in *Escherichia coli*. *Chinese journal of biotechnology*, *35*(1), 59-69. <https://doi.org/10.13345/j.cjb.180107>

Wei, X., Wu, J., Guo, P., Zhou, S., & Wu, H. (2021). Effect of short-chain thioesterase deficiency on P(3HB-*co*-LA) biosynthesis in *Escherichia coli*. *Chinese journal of biotechnology*, *37*(1), 196-206. <https://doi.org/10.13345/j.cjb.200243>
